# Constraints on novel word learning in heritage speakers

**DOI:** 10.3389/fpsyg.2024.1379736

**Published:** 2024-04-17

**Authors:** Yuxin Ge, Anabela Rato, Patrick Rebuschat, Padraic Monaghan

**Affiliations:** ^1^Linguistics and English Language, Lancaster University, Lancaster, United Kingdom; ^2^Linguistics Research Centre, NOVA University Lisbon, Lisbon, Portugal; ^3^Department of Spanish and Portuguese, University of Toronto, Toronto, ON, Canada; ^4^LEAD Graduate School and Research Network, University of Tübingen, Tübingen, Germany; ^5^Department of Psychology, Lancaster University, Lancaster, United Kingdom

**Keywords:** statistical learning, cross-situational word learning, heritage speaker, heritage language phonology, lexical tone

## Abstract

**Introduction:**

Recent research on word learning has found that adults can rapidly learn novel words by tracking cross-situational statistics, but learning is greatly influenced by the phonological properties of the words and by the native language of the speakers. Mandarin-native speakers could easily pick up novel words with Mandarin tones after a short exposure, but English-native speakers had specific difficulty with the tonal components. It is, however, unclear how much experience with Mandarin is needed to successfully use the tonal cue in word learning. In this study, we explored this question by focusing on the heritage language population, who typically are exposed to the target language at an early age but then develop and switch to another majority language. Specifically, we investigated whether heritage Mandarin speakers residing in an English-speaking region and speaking English as a dominant language would be able to learn novel Mandarin tonal words from statistical tracking. It helps us understand whether early exposure to the target feature is sufficient to promote the use of that feature in word learning later in life.

**Methods:**

We trained 30 heritage Mandarin speakers with Mandarin pseudowords via a cross-situational statistical word learning task (CSWL).

**Results and discussion:**

Heritage Mandarin speakers were able to learn the pseudowords across multiple situations, but similar-sounding words (i.e., minimal pairs) were more difficult to identify, and words that contrast only in lexical tones (i.e., Mandarin lexical tone) were distinguished at chance level throughout learning. We also collected information about the participants’ heritage language (HL) experience and usage. We did not observe a relationship between HL experience/usage and performance in tonal word learning, suggesting that HL exposure does not necessarily lead to an advantage in learning the target language.

## Introduction

Language learners can rapidly pick up new words from the surrounding environment, most of the time without explicit instruction. This is impressive given the highly variable environment in which language learning happens. Quine (1960) illustrated this word learning challenge by referring to the well-known “Gavagai” conundrum. The first time a learner encounters a new word, the meaning is usually unclear because the word could refer to anything in the environment. Without any explicit information, the word-referent mapping is ambiguous. How do learners deal with this referential ambiguity problem in real life?

Research on statistical learning has found a potential solution to the Gavagai problem: child and adult learners can keep track of the linguistic information across multiple situations to aid word learning, an ability commonly referred to as *cross-situational word learning* (CSWL; e.g., Suanda and Namy, 2012; Monaghan et al., 2019; Rebuschat et al., 2021; Escudero et al., 2022). That is, when the same word occurs again, learners can track the always-co-occurring referent and, over time, form an association between the word and the referent. However, recent studies have shown that CSWL is greatly influenced by the phonological properties of the words (Escudero et al., 2016; Tuninetti et al., 2020; Ge et al., 2024). Words that sound similar (e.g., phonological minimal pairs like *bag* vs. *beg* in English; *pāo* vs. *gāo* in Mandarin) generated difficulty in CSWL (e.g., Escudero et al., 2016), as well as the presence of non-native phonological features when adults learn an additional language (L2) via CSWL(e.g., Escudero et al., 2022; Ge et al., 2024; Ge et al., under review^1^). For example, L1 Mandarin speakers could learn Mandarin pseudowords from CSWL exposure regardless of the existence of tonal minimal pairs, but L1 English speakers had great difficulty with these non-native minimal pairs (Ge et al., 2024). This is because Mandarin-native speakers had extensive experience with the Mandarin tonal feature since childhood and could make use of the tonal categories in identifying words, but English-native speakers had no experience with tones and did not have the tonal representations. One question that arises is how much experience with the target feature would then be needed to develop the phonological representations and consequently use the feature in word learning.

To address this question, we targeted the heritage speaker population who are typically exposed to a minority (heritage) language at home in childhood, but start to rapidly acquire a different societal/majority language at the onset of school and become dominant in the societal/majority language. Specifically, we tested heritage speakers of Mandarin who were born to at least one Mandarin-speaking parent and resided in English-speaking countries from birth. These participants had early experience with the (Mandarin) tonal feature but then, later in life, had relatively limited use of lexical tones given that their majority language (English) is non-tonal. The performance of heritage speakers is particularly interesting because human sensitivity to sounds is largely shaped and tuned to their native languages at an early age, and hence experience with the target feature in early years might make a great difference even when exposure to the feature reduces later in life (Kuhl, 2004; Hartshorne et al., 2018). To summarize, in this study, we examined whether and how heritage speakers learn novel words from their heritage language (HL) via statistical tracking, and how they are affected by sounds that only exist in their HL but not in the majority language (i.e., lexical tones). Additionally, we tested whether the degree of HL experience and usage has an impact on word learning outcomes.

### Statistical word learning

Language learners can extract statistical regularities of different aspects of the language from the linguistic input (e.g., Maye and Gerken, 2000; Maye et al., 2002, for sound discrimination; Saffran et al., 1996, for word segmentation; see Siegelman, 2020; Isbilen and Christiansen, 2022; Williams and Rebuschat, 2022, for reviews). As for word learning, this involves tracking word-referent co-occurrences across encounters. A cross-situational statistical learning paradigm has often been used to examine word learning under implicit learning conditions where there is ambiguity in words’ referents (e.g., Yu and Smith, 2007; Smith and Yu, 2008; Suanda et al., 2014; Rebuschat et al., 2021; Escudero et al., 2022). For example, in Yu and Smith’s (2007) seminal study, adult learners were first presented with multiple words and pictures in each learning trial, and then tested whether they could make use of the word-picture co-occurrence information across learning events to acquire the appropriate mappings. After only 6 min of exposure, learners could match pictures to words at above-chance level even in highly ambiguous conditions where four words and four pictures were presented in each learning trial.

However, this rapid learning effect has been found to reduce when there are phonological overlaps between words, which can be found in most vocabulary inventories (e.g., Escudero et al., 2016, 2022; Tuninetti et al., 2020). For example, when being presented with two pictures and two minimal pair words in each learning trial, Escudero et al. (2016) reported that learners’ performance was inhibited—especially when the words were vowel minimal pairs (e.g., /dit/−/dɪt/)—compared to non-minimal pair presentations (e.g., /bɔn/−/dit/). This phonological similarity effect was even more profound when it came to L2 word learning. When the same CSWL task with English pseudo-minimal pairs (e.g., /dit/−/dɪt/, /bɔn/−/tɔn/) was presented to English-native and Mandarin-native speakers, it was observed that English-native speakers’ overall word learning performance was better than the Mandarin-native speakers in different minimal pair types (Escudero et al., 2022). Thus, the existence of non-native English contrasts influenced Mandarin-native speakers’ word learning outcomes. Similar evidence came from Australian English speakers learning Dutch and Brazilian Portuguese pseudo-minimal pairs (Tuninetti et al., 2020). Vowel minimal pairs were created based on Dutch and Brazilian Portuguese vowel inventories (e.g., /piχ/−/pyχ/, /fεfe/−/fefe/, respectively). As predicted, based on the Second Language Linguistic Perception model (L2LP—Escudero, 2005) and the Perceptual Assimilation-L2 model (PAM-L2—Best and Tyler, 2007), some of the vowel pairs were defined as perceptually easier as they could be mapped to two separate Australian English vowel categories (e.g., Dutch /i/−/ɑ/ contrast might be mapped to AusEnglish /i/−/ɔ/), and some other vowel pairs were classified as perceptually difficulty as they had no clear corresponding Australian English contrasts (e.g., Dutch /i/−/y/ contrast). Learners performed better with perceptually easy pairs compared to the difficult pairs, indicating that the degree of perceptual cross-linguistic similarity associated with non-native segments influenced non-native statistical word learning.

Ge et al. (2024) found that the non-native phonology effect in CSWL was not only associated with segmental but also suprasegmental features. In addition to the segmental minimal pairs as in previous research (e.g., Escudero et al., 2022), Ge et al. (2024) involved tonal minimal pairs (i.e., two words that differ only in lexical tone: /pa1mi1/ vs. /pa4mi1/ with numbers referring to Mandarin Tone 1 and Tone 4), which is a suprasegmental feature absent in non-tonal languages like English. A slightly different CSWL design is used to more closely resemble the minimal pairs learners encounter in the real world. Only one word was presented in each trial together with multiple referents, hence, minimal pairs were not presented side by side to participants in a single trial. This mirrors natural language learning situations in that minimal pairs tend not to occur in immediate proximity but need to be acquired by tracking the contrastive phonological features across situations. Through a short cross-situational exposure of 10 min, participants who were English-native speakers successfully identified word-referent mappings in consonantal, vocalic and non-minimal pairs, as the segmental features in the stimuli were designed to be familiar to English speakers, but not in the tonal pairs. Participants who were Mandarin-native speakers, on the other hand, were able to identify words in the tonal pairs after the same amount of exposure. These previous findings all suggest a significant role of phonology in statistical word learning and that L2 learners might encounter difficulty in picking up words from the environment because of the non-native sounds.

Such difficulty has been found even when specific phonetic (perceptual) training on the target non-native contrasts is included (Ge et al., under review) (See footnote 1). For example, in Ge et al., under review (See footnote 1), native speakers of English were provided with perceptual training on Portuguese consonant and vowel contrasts (e.g., /l/−/ʎ/, /n/−/ɲ/, /e/−/ɛ/, /o/−/ɔ/), and then trained on Portuguese pseudowords containing these contrasts via CSWL. The perceptual training did improve learners’ perceptual discrimination of the non-native contrasts, but this improvement did not transfer to word learning – the English-native speakers still had difficulty with non-native minimal pairs in word learning. This finding indicates that L2 learners’ difficulty comes from not simply perceptual issues, but also the lack of phonological representation of the novel sounds. As widely reported in infant speech development literature, during as early as the first year of life, humans start to tune in to their native sound system(s) and their sensitivity to non-native sounds and categories greatly reduces (e.g., Werker and Tees, 1984; Kuhl, 2004; Watson et al., 2014). This perceptual tuning persists into adulthood and might contribute to the difficulties in L2 word learning. Previous studies observed a phonetic-phonological-lexical continuity, indicating that categorical perception of non-native sounds was associated with performance in non-native word learning and processing (e.g., Wong and Perrachione, 2007; Ling and Grüter, 2022; Laméris et al., 2023). Hence, if the narrowing process in early years does play a significant role, one question that follows is whether exposure to the target language in early years would facilitate word learning (in the same language) later in life, as early exposure might allow learners to develop the necessary perceptual sensitivities and phonological categories.

A particular population that is perfect to study this research question is heritage speakers because of their special language profile. Like all native speakers of a language, heritage speakers have early exposure to the language, which would allow them to develop sensitivities to the language-specific phonological contrasts, but they switch to another dominant language after the early years and usually have limited HL use afterwards. It thus allows us to specifically test whether early exposure to the target language plays a role in later word learning. In other words, we explored whether heritage speakers’ phonological representations that are developed early in life remain accessible and help them learn new words from their HL in adulthood.

### Phonological advantages in heritage speakers

HL research has observed phonological advantages among heritage speakers in both speech perception and production compared to late L2 learners, and closer performance to native speakers in some dimensions (e.g., Lukyanchenko and Gor, 2011; Chang, 2016, for speech perception; Au et al., 2002; Chang et al., 2011, for speech production; Flores et al., 2017, for accentedness). For example, heritage Korean speakers who grew up in an English-speaking environment showed greater sensitivity to unreleased stops as it is an obligatory feature in Korean (Chang, 2016). Although unreleased final stops are present in American English, it is not considered the canonical form and English speakers rely more on released stops in word recognition. It was found that heritage Korean speakers’ identification of the unreleased stops (in Korean and English) was comparable to L1 Korean speakers and was better than L1 English speakers. This suggests that early exposure to the phonological contrasts did persist into adulthood and facilitate sound recognition later in life. As for speech production, for instance, Chang et al. (2010) reported that compared to L2 Mandarin learners, heritage Mandarin speakers’ back vowel production (e.g., Mandarin /u/) was closer to native Mandarin speakers (though not the same). In addition to the segmental features, some research also found an advantageous performance in heritage speakers’ suprasegmental realizations (e.g., Yang, 2015; Chang and Yao, 2016, 2019, for lexical tone; Kim, 2020, for lexical stress). Regarding lexical tone, for example, Yang (2015) examined the perception and production of Mandarin tones by native Mandarin speakers, heritage Mandarin speakers, and L2 learners. Heritage speakers’ perception of tones lay in between the native and the L2 groups: heritage speakers exhibited a more stable categorical perception of the four tones than L2 learners, although they do not completely resemble native Mandarin speakers’ perceptual patterns. Work on Mandarin speech production showed that heritage Mandarin speakers’ production of tones also fell in the intermediate state between native and L2 speakers in general (Chang and Yao, 2016). In some dimensions, heritage speakers’ tonal production resembles more native speakers (e.g., T3 low falling-rising tone turning point), whereas in some other dimensions, heritage speakers’ production was in between the native and L2 groups (e.g., tone shortening in multisyllabic contexts). Overall, although heritage speakers do not pattern exactly the same as native speakers, much research evidence has shown that they are at least closer to native speakers in terms of speech perception and production than L2 learners are.

However, it is not clear if heritage speakers can make use of such phonological advantages at the lexical level to assist novel word learning in the HL. As discussed in the previous section, phonologically similar words pose difficulties for L2 learners when they lack the appropriate phonological representations. Here, we hypothesize that heritage speakers’ advantages in speech perception and recognition would further facilitate their acquisition of phonologically overlapping words in the target language. In this study, we focus on a suprasegmental feature that has been found to be difficult for late L2 learners in word learning—lexical tones (Ge et al., 2024). L2 learners of Mandarin were found to fail in learning tonal minimal pair words from implicit exposure, whereas L1 Mandarin speakers could pick up novel tonal minimal pairs rapidly in the same situation. Our prediction is that heritage Mandarin speakers would be able to learn tonal minimal pairs to some extent because of their better categorical tonal perception, but whether they could match native speakers’ performance largely depends on their individual HL experience.

### Research questions and predictions

In the current study, we investigate the cross-situational learning of Mandarin pseudowords by adult heritage speakers of Mandarin who were born and reside in English-speaking countries. The following research questions are addressed:

RQ1: Do minimal pairs and phonological contrasts that do not exist in heritage speakers’ majority language (i.e., the tonal contrasts) pose difficulty during cross-situational learning?

RQ2: Does the degree of heritage language experience and usage influence learning outcomes?

For RQ1, based on previous literature, we predicted that minimal pairs would be more difficult to learn compared to non-minimal pairs, and minimal pairs with phonological contrasts that do not exist in heritage speakers’ dominant language would generate the greatest difficulty in learning (Escudero et al., 2022; Ge et al., 2024). Specifically, we predicted that minimal pairs that contrast in lexical tones would be the most difficult (i.e., with the lowest accuracy), followed by minimal pairs that differ in consonants and vowels. The non-minimal pairs would be relatively easy to learn. However, we expected the heritage Mandarin speakers to show some degree of learning of the tonal minimal pairs.

For RQ2, we predicted that greater experience and usage of HL would be associated with better learning of the tonal minimal pairs, as participants with greater Mandarin experience and usage would have more exposure to the tonal contrasts and might be more sensitive to the tonal minimal pairs.

## Methods

### Participants

Thirty bilingual speakers of Mandarin Chinese and English participated in this study. The sample size was inferred from Ge et al. (2024),^2^ where the same stimuli and CSWL task were used and a significant learning effect was observed. Participants were recruited through email advertisements within university communities in Toronto, Canada, and through Prolific.^3^ Participants had to be at least 18 years old, bilingual speakers of English and Mandarin Chinese, and born in an English-speaking country (Canada or United States). An additional prerequisite was that participants needed to have at least one parent who was a native speaker of Mandarin Chinese. One participant was excluded because they were born in Hong Kong and only moved to an English-speaking country at the age of four. Thus, 29 participants were included in the data analysis (11 F, 17 M 1 preferred not to say). The mean age was 29.97 (SD = 8.60, ranging from 18 to 62 years). Regarding language background, 14 participants reported knowing additional languages/varieties other than Mandarin or English (e.g., Cantonese,^4^ French, Italian, Shanghainese, and Spanish). Nine participants reported having one Mandarin-native parent, and 20 participants with two Mandarin-native parents. Further details on participants’ HL experience and use can be found in the results section.

### Materials

#### Heritage language experience questionnaire

We collected information about participants’ HL (i.e., Mandarin) experience using Tomić et al.’s (2023) Heritage Language Experience Questionnaire (HeLEx). The questionnaire was designed to capture the quantity and quality of HL exposure and use in different social contexts (e.g., family, external family (i.e., family outside the household), work, community, leisure). It also asked for participants’ background information (e.g., gender, age, history of language learning, parents’ language) and educational information (e.g., language used at different levels of schooling). Additionally, there were questions regarding participants’ language attitudes and code-switching attitudes and behaviors, though we did not include these attitude-related questions in the analyses because language attitude is not the focus of the current study.

For the HeLEx data, we followed Tomić et al.’s (2023) instructions and derived a set of HL experience and usage measures, including HL experience (i.e., frequency of use) and proficiency^5^ in four different modalities (reading, writing, speaking, listening), proportion of HL use in different social contexts (family, external family, work, community, leisure), language dominance, language entropy,^6^ proportion of HL use when accounting for actual time spent in each context (i.e., weighted HL use), and diversity of HL interlocutors (i.e., proportion of HL proficient and/or dominant interlocutors).

#### Cross-situational word learning task

The CSWL task involved 12 pseudowords and 12 referent pictures. All pseudowords were disyllabic, with CVCV structures, which satisfies the phonotactic constraints of both Mandarin Chinese and English. The pseudowords contained phonemes that were similar between the two languages. The choice of the phonotactics and phonemes ensured that the target feature, lexical tone, was the only feature that exist in participants’ heritage language but not in the majority language. Each syllable in the pseudowords carried a lexical tone which was either Tone 1 (high-level) or Tone 4 (high-falling) in Mandarin Chinese, thus creating a simplified lexical tone system.

Six consonants /p, t, k, l, m, f/ and four vowels /a, i, u, ei/ were combined to form eight distinct base syllables (/pa, ta, ka, li, lu, lei, mi, fa/), which were further paired to form six minimally distinct base words (/pami, tami, kami, lifa, lufa, leifa/). Three of the base pseudowords differed in the consonant of the first syllable (/pami, tami, kami/) and the other three differed in the vowel of the first syllable (/lifa, lufa, leifa/). These base words were then superimposed with lexical tones. The first syllable of each of the six base words was paired with either T1 or T4, and the second syllable always carried T1. This created additional tonal minimal pair contrasts (e.g., /pa1mi1*/* vs. /pa4mi1*/*). Therefore, a total of 12 pseudowords were created (full list shown in Table 1). The pseudowords (with their corresponding referent objects) were later paired to create consonantal, vocalic, tonal, and non-minimal pair trials, and each pseudoword-referent mapping could occur in different trial types based on the paired foil. All pseudowords had no corresponding meanings in English or Mandarin Chinese. The audio stimuli were produced by a female native speaker of Mandarin Chinese. The mean length of the audio stimuli was 800 ms.

**Table 1:** Pseudowords in the consonant set and the vocalic set.

Consonant set		Vocalic set	
pa1mi1	pa4mi1	li1fa1	li4fa1
ta1mi1	ta4mi1	lu1fa1	lu4fa1
ka1mi1	ka4mi1	lei1fa1	lei4fa1

Numbers “1” and “4” refer to the lexical tones T1 and T4 carried by the syllables.

Twelve pictures of novel objects were selected from Horst and Hout’s (2016) NOUN database and used as referents. The pseudowords were randomly mapped to the objects, and we created four lists of word-referent mappings to minimize the influence of a particular mapping being easily memorisable. Each participant was randomly assigned to one of the mappings.

The visual and auditory stimuli are available at: https://osf.io/q6354/.

### Procedure

All participants were directed to the experiment platform Gorilla^7^ to complete the task and the questionnaire. After providing informed consent, participants completed the CSWL task, which took approximately 10 min. In the CSWL task, participants were told that they would hear one word and see two pictures of referent objects on the screen. Their task was to decide, as quickly and accurately as possible, which object the pseudoword referred to. They were instructed to press ‘Q’ on the keyboard if they thought the object on the left was the correct referent of the word and ‘P’ for the object on the right.

**Figure 1:**
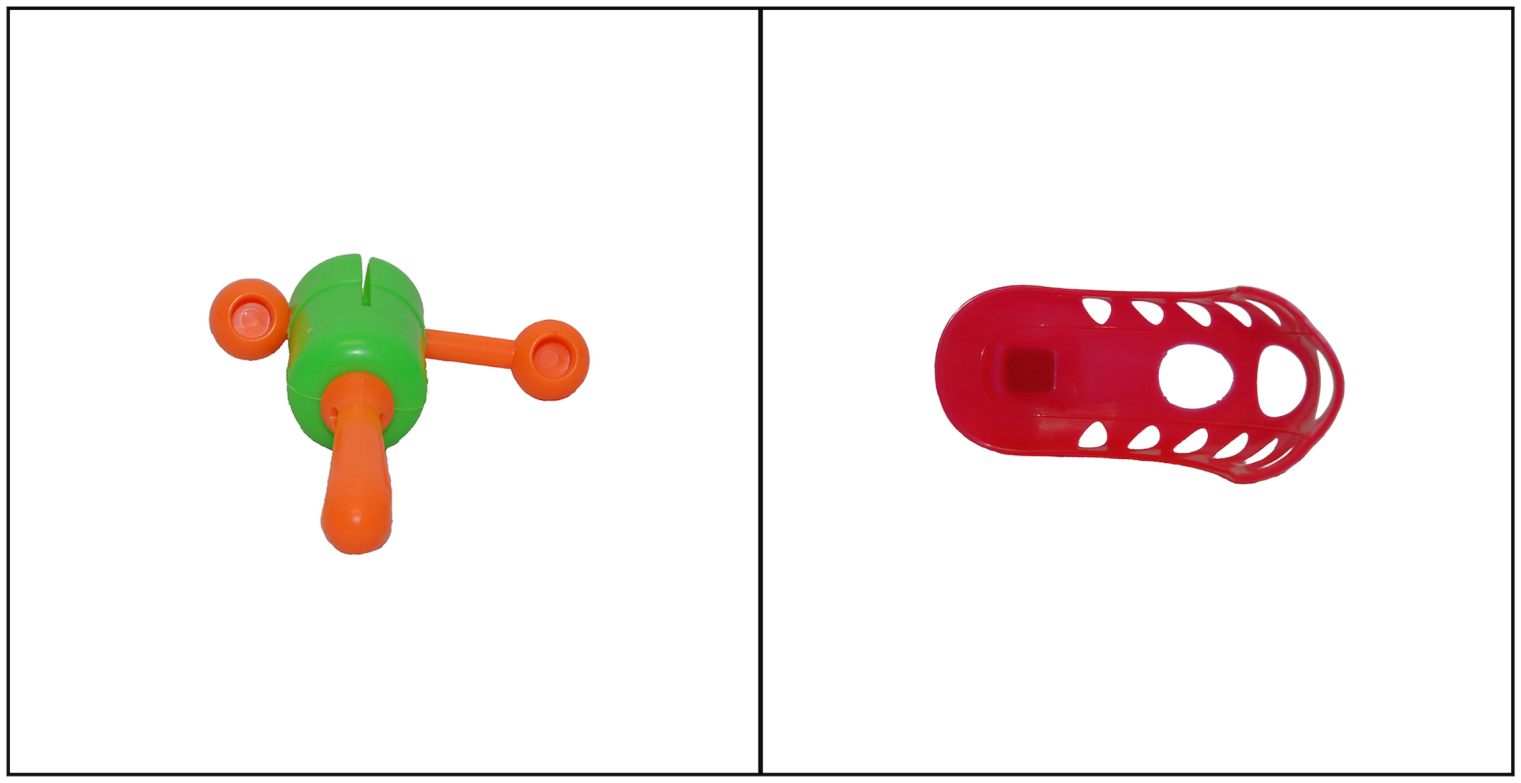
Example of cross-situational word learning (CSWL) trial. Participants were presented with two objects and played a single pseudoword. They had to decide if the pseudoword referred to the object on the left or the object on the right.

In each trial, participants first saw a fixation cross at the centre of the screen for 500 ms. They were then presented with two objects on the screen (one on the left side and one on the right) and were played a single pseudoword. After the pseudoword was played, participants were prompted to enter their response on the keyboard (Q or P). The objects remained on the screen during the entire trial, but the pseudoword was only played once. The next trial only started after participants made a choice for the current one. No feedback was provided after each response. We recorded the keyboard responses in each trial to calculate accuracy and response times. This allowed us to keep track of participants’ performance throughout the CSWL task, and hence there were no separate training and testing phases. Figure 1 provides an example of a CSWL trial.

There were four types of CSWL trials. In non-minimal pair (non-MP) trials, the two objects presented on the screen referred to pseudowords that were phonologically distinct (e.g., /pa1mi1/ and /li4fa1/). In consonantal minimal pair (cMP) trials, the two objects on the screen referred to pseudowords that differed in only one consonant contrast (e.g., /pa1mi1/ and /ta1mi1/). In vocalic minimal pair (vMP) trials, the two objects referred to pseudowords that differed in only one vowel contrast (e.g., /li1fa1/ and /lu1fa1/). And in tonal minimal pair (tMP) trials, the two objects referred to pseudowords that differed only in lexical tone (e.g., /pa1mi1/ and /pa4mi1/). This manipulation allowed us to determine if and how phonological overlap between the pseudowords affected word learning. Each object was paired with different foils according to the trial type. For instance, the object for *pa1mi1* was paired with the (foil) object for *ta1mi1* in a consonantal minimal pair trial; and the same object for *pa1mi1* was paired with the (foil) object for *pa4mi1* in a tonal minimal pair trial.

Each participant completed six CSWL blocks, with each pseudoword-object mapping occurring twice per block. There were thus 24 trials per block, and 144 trials in total. The four trial types (non-MP, cMP, vMP, tMP) occurred six times per block. The order of trials within each block was randomized for each participant as was the sequence in which the six blocks occurred. The correct referent picture was presented on the left side in half of the trials and on the right side in the other half of the trials.

After the CSWL task, participants completed the HeLEx questionnaire. When all tasks were completed, participants recruited from Prolific were directed back to the Prolific website and were granted compensation. Participants recruited through emailing received the vouchers via email.

### Data analysis

We excluded participants who failed to successfully complete the initial sound check (one participant failed, and 30 participants passed the sound check). We also excluded individual responses that lasted over 30 s (11 out of 4,176 individual responses were removed, leaving a total of 4,165 data points for analysis). This was because they failed to follow the instruction to respond as quickly and accurately as possible. After excluding these data points, we visualized the data using R (R Core Team, 2022) for general descriptive patterns. We then used generalized linear mixed effects modeling for statistical data analysis. Mixed effects models were constructed from the null model (containing only random effects of item and participant) to models containing fixed effects, and the dependent variable was accuracy in the CSWL task. We tested if each of the fixed effects of trial type, block, and their interaction improved model fit using log-likelihood comparisons between models. A quadratic effect of block was also tested for its contribution to model fit, as learning may have been non-linear over training. Additionally, we tested if adding the derived measures from the HeLEx questionnaire as fixed effect to the mixed-effect models improved model fit.

The anonymized data and R scripts are available at: https://osf.io/q6354/.

## Results

### Performance on the cross-situational word learning task

**Figure 2:**
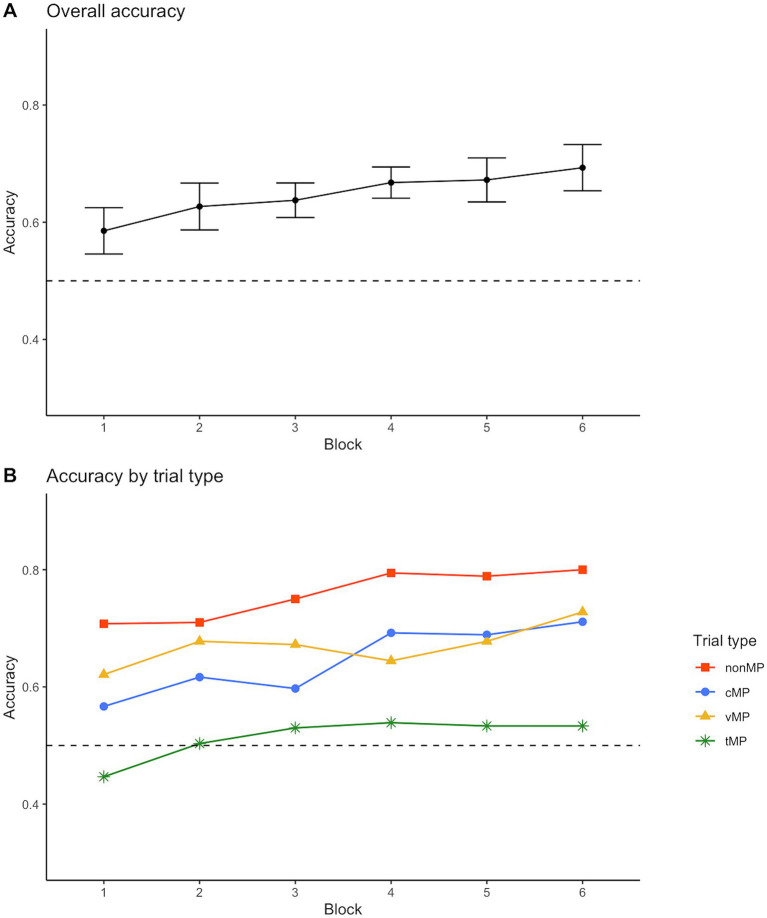
Mean proportion of correct pictures selected in each learning block—overall **(A)** and in different trial types **(B)**. The dotted line represents chance level. Error bars represent 95% confidence intervals.

Figure 2A presents the overall proportion of correct responses in the CSWL task. Participants performed significantly above chance from Block 1 (mean accuracy = 0.59, *t* = 4.61, *p* < 0.001). For the different minimal pair trials (Figure 2B), accuracy was the highest in non-minimal pair trials, followed by consonantal and vocalic minimal pair trials. Performance in the tonal trials was the lowest and remained close to chance level (0.53) until the end of the CSWL task.

We ran generalized linear mixed effects models to examine performance accuracy across learning blocks. Compared to the model with only random effects, adding the fixed effect of learning block did not improve model fit significantly (χ^2^(1) = 0.944, *p* = 0.331). Adding trial type (consonant, vowel, tone, non-minimal pair) improved model fit (χ^2^(3) = 28.298, *p* < 0.001), but the block*trial type interaction (χ^2^(3) = 4.365, *p* = 0.225) did not improve fit further. This indicates that the overall performance differed significantly across trial types, but the learning trajectories (i.e., improvement across blocks) did not differ significantly in different trial types. The quadratic effect for block did not result in a significant difference (χ^2^(4) = 2.109, *p* = 0.716). The best-fitting model is reported in Table 2. Note that, whereas block did not contribute to explaining variance significantly when considered as a single fixed effect, it was significant in the model when trial type was also included (as shown in Table 2).

**Table 2:** Best fitting model for accuracy in CSWL, showing fixed effects.

Fixed effects	Estimate	SD error	Z	*p*-value
(Intercept)	0.934	0.176	5.298	< 0.001***
Block	0.105	0.031	3.399	< 0.001***
TrialTypeC	−0.604	0.138	−4.383	< 0.001***
TrialTypeT	−1.217	0.179	−6.803	< 0.001***
TrialTypeV	−0.454	0.148	−3.078	0.002**

TrialTypeC refers to consonantal minimal pair trials, TrialTypeT refers to tonal minimal pair trials, TrialTypeV refers to vocalic minimal pair trials, with the reference being non-minimal pair trials.

Number of observations: 4165, Participants: 29, Item, 12. AIC = 5076.1, BIC = 5297.8, log-likelihood = −2503.1.

R syntax: glmer[acc ~ block + TrialType + (1 + block + TrialType | item) + (1 + block + TrialType | subjectID), family = binomial, data = fulld, glmerControl(optCtrl = list(maxfun = 2e5), optimizer = "nloptwrap," calc.derivs = FALSE)]. **p < 0.01; ***p < 0.001.

### Heritage language experience questionnaire

We computed a set of measures of HL use derived from the four modalities (reading, writing, speaking, hearing) and five contexts (family, external family, work, community, leisure) of language use. Tables 3, 4 summarize the results.

**Table 3:** Heritage language experience across four modalities.

	Reading	Writing	Speaking	Hearing	Scale
HL experience	3.97 (2.01)	2.97 (2.11)	5.48 (1.33)	5.83 (1.26)	1 ~ 7
HL proficiency	2.14 (0.95)	1.93 (0.96)	2.86 (0.64)	3.34 (0.67)	1 ~ 4
HL/SL dominance (experience-based)	0.57 (0.29)	0.43 (0.30)	0.79 (0.19)	0.83 (0.17)	1 = balanced Mandarin and English
HL/SL dominance (proficiency-based)	0.53 (0.24)	0.49 (0.24)	0.75 (0.20)	0.85 (0.18)	

**Table 4:** Heritage language (Mandarin) use in five contexts.

	Family	External family	Work	Community	Leisure
Proportion of HL use	0.64 (0.28)	0.64 (0.29)	0.10 (0.21)	0.12 (0.13)	0.18 (0.22)
Proportion of HL interaction	0.43 (0.28)	0.39 (0.34)	0.07 (0.17)	0.10 (0.20)	0.13 (0.23)
Proportion of HL use (weighted)	0.30 (0.22)	0.06 (0.08)	0.03 (0.08)	0.01 (0.02)	0.02 (0.04)
Proportion of HL proficient interlocutors	0.80 (0.31)	0.63 (0.46)	0.22 (0.42)	0.30 (0.47)	0.33 (0.46)
Proportion of HL dominant interlocutors	0.72 (0.36)	0.54 (0.46)	0.20 (0.39)	0.29 (0.46)	0.25 (0.42)
Language entropy	0.67 (0.34)	0.64 (0.37)	0.25 (0.30)	0.41 (0.34)	0.46 (0.39)

Participants reported higher Mandarin proficiency and use in speaking and hearing compared to reading and writing. As for language dominancy, only one participant reported to be Mandarin-dominant in speaking and another participant being Mandarin-dominant in hearing/understanding. Overall, more participants were dominant in English in all modalities. In terms of the context of language use, participants reported more Mandarin use with families and external families, and relatively little Mandarin use in working conditions.

### The relationship between heritage language background and CSWL

To investigate whether the proficiency and use of Mandarin influence the outcomes in learning novel tonal words (i.e., performance at the final block), we ran several sets of mixed-effect models with the derived measures from HeLEx as fixed effects.

For the measure of Mandarin use across modalities, we carried out three sets of analyses to explore the fixed effects of (1) Mandarin proficiency, (2) frequency of Mandarin usage, (3) usage-based and proficiency-based Mandarin dominance in the four modalities. ANOVA comparison between models containing fixed effects and the random effect model showed no significant differences, indicating that none of these fixed effects significantly explain variance in word learning outcomes.

As for the measures of Mandarin use in the five contexts, we ran four sets of analyses and tested if (1) the proportion of Mandarin use, (2) the proportion of Mandarin interaction, (3) language entropy, (4) the weighted proportion of Mandarin use (accounting for the actual time spent in each context) in the different contexts explained performance in the tonal trials. However, we did not find any significant predictors of performance from the derived measures.

#### Exploratory analyses

Since we did not observe any significant influence of the individual HeLEx measures on participants’ learning outcomes in tonal trials, we carried out additional exploratory analyses based on other responses in the questionnaire. Firstly, we explored if having one or two Mandarin-native parent influences learners’ performance, as having two Mandarin-native parents may provide a more Mandarin-dominant environment at home. Mixed-effects models containing parent language as a fixed effect showed no significant improvement compared to the random effect model (χ^2^(1) = 0.0801, *p* = 0.78). This means that the number of Mandarin-speaking parent did not explain variance in word learning outcome. Secondly, we coded whether or not participants used Mandarin at preschool, primary school, secondary school, post-secondary and post-graduate levels, and extracurricular Mandarin classes to test the effect of Mandarin schooling. Model comparisons revealed no significant effect of any of the variables.

#### Exploratory factor analysis

Given the large number of observed variables derived from the questionnaire, we decided to carry out an exploratory factor analysis and examine whether some of the variables could be grouped into a smaller number of factors for further analyses. We planned to run two rounds of factor analysis, one for the modality-related variables (see Table 3) and another for context-related variables (see Table 4). This is because mixing the variables across modalities and the variables across contexts might make the resulting factors less interpretable.

For the modality-related variables, we first checked the correlations between HL experience and experience-based dominance measures, as well as between HL proficiency and proficiency-based dominance measures. The results suggested that the measures are very strongly correlated (*r* > 0.90), which was expected because they were derived from the same set of original questions. Thus, we took out the dominance measures and only entered HL experience and HL proficiency across modalities into the factor analysis. The exploratory factor analysis suggested three factors: Factor 1 relates to measures of written language experience and proficiency (i.e., reading/writing experience, reading/writing proficiency), Factor 2 relates to measures of oral language experience (i.e., speaking/hearing experience), and Factor 3 relates to measures of oral language proficiency (i.e., speaking/hearing proficiency). Table 5 summarizes the output factor loadings of each measure.

**Table 5:** Factor loadings for modality-related variables.

	Factor 1 (reading and writing)	Factor 2 (oral experience)	Factor 3 (oral proficiency)
Reading_Experience	0.741		
Writing_Experience	0.861		
Reading_Proficiency	0.869		
Writing_Proficiency	1.005		
Speaking_Experience		0.911	
Hearing_Experience		0.996	
Speaking_Proficiency			0.933
Hearing_Proficiency			0.676

We then entered the three factors as fixed effects into the generalized mixed effect models mentioned above to explore if the grouped factors predicted participants’ learning outcomes. Similar to our previous findings, ANOVA comparisons between models containing fixed effects of the three factors and the random effect model showed no significant differences, meaning that the three modality-related factors did not significantly explain variance in word learning outcomes.

**Figure 3:**
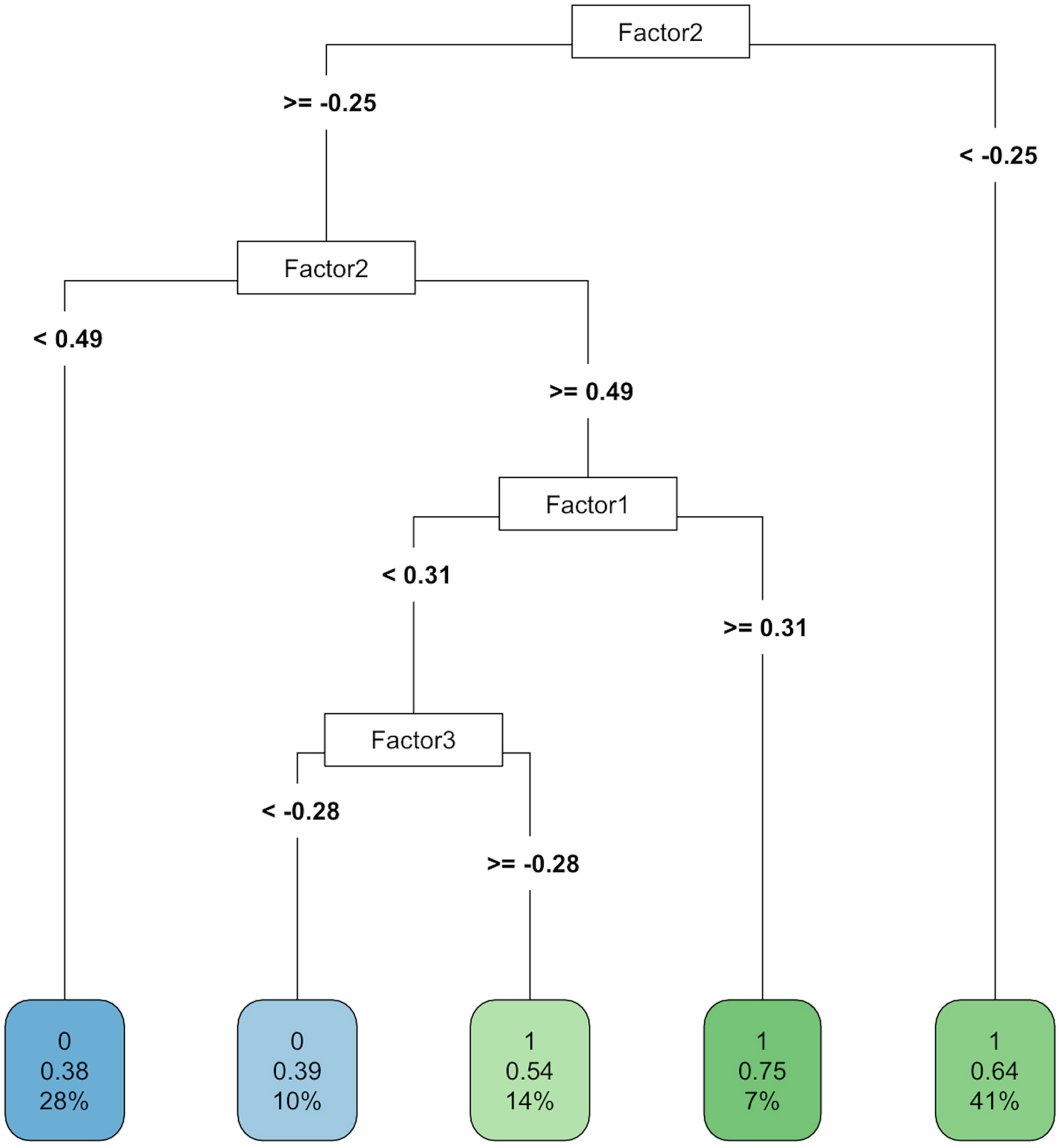
Decision tree model based on the three modality-related factors.

In addition, we ran a decision tree analysis to explore and visualize the hierarchical contribution of the three factors to word learning outcomes. Figure 3 presents the results of the decision tree model. Higher Factor 2 score (oral experience) and Factor 1 score (written experience and proficiency) seemed to lead to a path to higher accuracy in tonal trials at the final block (when Factor 2 > = 0.49 and Factor 1 > = 0.31, accuracy = 0.75), though only a small proportion of data fell under this rule. Overall, however, the decision tree model did not provide clear relations between the factors and the tonal word learning outcomes.

We then tried to fit the same factor analysis and follow-up tests on the context-related measures. However, there was no good factor solution for the context-related measures (Kaiser-Meyer-Olkin test suggested that data was not suitable for factor analysis)—indicating that the individual measures of context of use should be kept separate. Thus, no further analyses based on the derived factors were conducted.

### Comparison with English-native and Mandarin-native participants

To further understand Mandarin heritage speakers’ word learning performance, we ran exploratory analyses combining data from the current study and data from Ge et al. (2024) since the two studies employed the same method and stimuli. This allowed us to compare Mandarin heritage speakers’ learning trajectory with English-native participants (who had no tonal experience) and Mandarin-native participants (who had continuous, extensive tonal experience). Generalized linear mixed effects models revealed that, compared to the model with only random effects, adding the fixed effect of block (χ^2^(1) = 21.012, *p* < 0.001), trial type (χ^2^(3) = 28.532, *p* < 0.001), and the 3-way block*trial type*language group interaction (χ^2^(11) = 42.459, *p* < 0.001) significantly improve model fit. The effect of language group (English-native, Mandarin-native, Mandarin heritage) did not improve fit (χ^2^(2) = 0.824, *p* = 0.662).

We then explored the 3-way interaction in detail and ran separate mixed effects models for each trial type to test whether the group performances differed in any particular trial types. In the tonal trials, we observed a significant effect of language group (χ^2^(2) = 6.851, *p* = 0.033). The effect of block (χ^2^(1) = 3.386, *p* = 0.066) and the block*language group interaction (χ^2^(2) = 0.020, *p* = 0.990) was not significant. The best-fitting model summarized in Table 6 shows that the Mandarin-native group performed significantly better than the English-native group (the reference group) in tonal trials, whereas the Mandarin heritage group did not show significant divergence from the English-native group. This language group effect, however, was not significant in other trial types (consonantal χ^2^(2) = 3.370, *p* = 0.185; vocalic χ^2^(2) = 2.254, *p* = 0.324; non-minimal pair χ^2^(2) = 3.149, *p* = 0.207).

**Table 6:** Best fitting model for accuracy in tonal trials, combining data from the present study and data from Ge et al. (2024).

Fixed Effects	Estimate	SD Error	Z	*p*-value
(Intercept)	−0.188	0.143	−1.314	0.189
Block	0.061	0.026	2.345	0.019 *
GroupMandarinL1	0.451	0.170	2.657	0.008 **
GroupMandarinHeritage	0.066	0.116	0.569	0.570

Number of observations: 3049, Participants: 85, Item, 12. AIC = 4186.6, BIC = 4355.2, log-likelihood = −2065.3.

R syntax: glmer[acc ~ block + langgroup + (1 + block + langgroup + block:langgroup | item) + (1 + block | subjectID), family = binomial, data = fulld.combined, glmerControl(optCtrl = list(maxfun = 2e5), optimizer = "nloptwrap," calc.derivs = FALSE)]. *p < 0.05; **p < 0.01.

## Discussion

In this study, we explored how heritage speakers learn novel words from their HL via a cross-situational, statistical learning process and whether the degree of HL experience predicts learning outcomes. Heritage speakers could rapidly learn words that contain special phonological features which exist only in their HL but not in their dominant language (i.e., lexical tone for heritage Mandarin speakers residing in English-speaking environments). However, when this specific feature is the only informative cue to distinguish words (i.e., in the case of tonal minimal pairs), heritage speakers seem to encounter greater difficulties.

*RQ1: Do minimal pairs and phonological contrasts that do not exist in heritage speakers’ majority language pose difficulty during cross-situational learning?*

Results suggested that learners’ performance was greatly influenced by the presence of minimal pair words. As predicted, learners performed better in non-minimal pair trials as compared to minimal pair trials, which is consistent with previous findings on CSWL of minimal pairs in other languages (e.g., Escudero et al., 2022). Moreover, we observed a difference in performance on segmental minimal pairs and tonal minimal pairs. Heritage Mandarin speakers’ performance in tonal minimal pair trials was the lowest and remained at chance level throughout the experiment, whereas performance in consonantal and vocalic minimal pair trials improved over time. The lack of learning effect in tonal trials was contrary to our prediction that early exposure to Mandarin would allow the heritage speakers to develop tonal representations and be able to use tonal cues in word learning. Our combined data analysis with Ge et al. (2024) demonstrated that the Mandarin heritage speakers’ learning pattern was similar to English-native speakers with no tonal experience, where tonal minimal pairs were particularly difficult, and performance in tonal trials was significantly lower than that of Mandarin-native speakers.

These findings could be explained from two perspectives – the nature of the stimuli and the participants’ language profile. Firstly, the stimuli in the experiment were designed to have segments that are similar between English (the dominant language) and Mandarin (the heritage language), and also include a tonal feature that is specific to Mandarin. Since our participants were English-dominant, they might weigh more the segmental cues in their linguistic repertoire and attend more to the segmental features in the task. Previous research also suggested that even Mandarin-native speakers tend to rely more on segmental than tonal information in word processing (e.g., Cutler and Chen, 1997; Yip, 2001; Sereno and Lee, 2015). This might contribute to the divergence in the learning trajectories of segmental and tonal minimal pairs. Secondly, although the group of heritage speakers we recruited reported relatively high proficiency in Mandarin listening (rating 3.34 out of 4) and speaking (rating 2.86 out of 4), they were still significantly more dominant in English in all language modalities (see Table 3, HL dominance), and had very little Mandarin use outside of the family (including external family) context (see Table 4). This might explain why their performance in the learning task at the group level resembles that of the English-native speakers in previous research (Ge et al., 2024).

Furthermore, considering previous findings on heritage Mandarin speakers’ perception and production of Mandarin tones (e.g., Chang and Yao, 2016, 2019), there is another possibility that derives from heritage speakers’ distinct tonal representations. Although heritage speakers of Mandarin tend to possess categorical representations of tones that are closer to native Mandarin speakers, they are usually not entirely the same as native speakers (e.g., Yang, 2015). Therefore, even though the heritage Mandarin speakers in the experiment possess sensitivity to tonal variations, their categorization of the specific contrast (i.e., T1–T4) might be different from the native speakers in certain acoustic dimensions, resulting in the difficulty in tonal minimal pair learning. Additionally, the selection of the tones used in the stimuli was based on previous experiment testing English-native speakers’ identification of Mandarin tones. Hao (2018) reported that English-native learners of Mandarin could identify T1 and T4 at word-initial positions better compared to T2 and T3, and hence these tones are likely to be easier in the disyllabic environment of this experiment. However, it is possible that the identification difficulty of the tones is different for heritage Mandarin speakers. Further research is needed to examine how tonal contexts (the preceding and following tones) affect heritage speakers’ perception in particular.

*RQ2: Does the degree of heritage language experience and usage influence learning outcomes?*

According to the HeLEx questionnaire results, we did not find a clear relationship between participants’ Mandarin experience or usage and their performance in the tonal word learning task. Specifically, the derived measures from the questionnaire did not predict how well participants respond to tonal minimal pairs. The questionnaire measures focused on how much and how well participants use Mandarin in their daily communications, that is, the use of Mandarin in various contexts. When using Mandarin for communicative purposes, lexical tones are not the only focus because information from the context can be delivered even when lexical tones are not always correctly realized. However, in the word learning task, there was no contextual information and participants had to learn isolated words. For the tonal minimal pair trials in particular, a misperception of lexical tone would lead to failure in word identification. It is possible that heritage Mandarin speakers might rely more on contextual information in tonal perception than native speakers. Thus, a direct link between the questionnaire measures and the word learning outcomes was missing because they measured tonal abilities in different communicative situations.

Another noteworthy finding is that our factor analysis suggested a grouping of the derived measures of HL modality use, highlighting a distinction between written and oral language proficiency and use. Questionnaires like HeLEx usually contain a large number of measures to thoroughly record participants’ language profiles. Our results suggested that some individual measures (even across the original categories) could be highly correlated and hence reasonably grouped into one single factor to facilitate further statistical analyses and predictions of the influence of HL on learning and behavior.

### Limitations and further directions

In the CSWL task, learning performance reflects the combined abilities at both the perceptual and lexical levels. Since we do not have a separate measure of tonal perception, it is unclear whether the difficulty comes from heritage Mandarin speakers’ different tonal representations and categorizations. Thus, further studies could add tone identification tasks to examine whether more accurate identification would be associated with better word learning. It would also be interesting to test tone identification at both the pre-lexical level (e.g., identification of isolated tonal syllables without meaning) and the lexical level (e.g., identification of tones in real words), since it indicates how well participants process tonal information when meanings are attached. Moreover, it would be worth testing whether greater HL experience and usage is directly linked to better tone identification ability.

Furthermore, it would be interesting to recruit participants from more diverse HL backgrounds. In our current sample, most participants were highly English-dominant. Future studies could compare whether heritage speakers who are more balanced in their English and Mandarin proficiency would perform differently and be more able to learn the tonal minimal pairs.

## Conclusion

We found that heritage speakers of Mandarin learned Mandarin novel words in a similar pattern to English-native learners of Mandarin. They could pick up new words from a short exposure by tracking the statistics of input, but learning was reduced when minimal pairs were present. The greatest difficulty was associated with tonal minimal pairs. The degree of HL experience and usage did not seem to predict tonal word learning outcomes. Our results contribute to the understanding of heritage speakers’ behaviors when learning and processing the target language. It suggests that heritage exposure does not necessarily lead to an advantage in learning the target language, and the amount of exposure may not be the key factor influencing learning outcomes, though further research into the role of diverse HL exposure is needed.

## Data availability statement

The datasets presented in this study can be found in online repositories. The names of the repository/repositories and accession number(s) can be found at: Open Science Framework (OSF): https://osf.io/q6354/.

## Ethics statement

The studies involving humans were approved by Faculty of Arts and Social Sciences and Lancaster Management School’s Research Ethics Committee Lancaster University. The studies were conducted in accordance with the local legislation and institutional requirements. The participants provided their written informed consent to participate in this study.

## Author contributions

YG: Conceptualization, Formal analysis, Methodology, Writing – original draft. AR: Conceptualization, Methodology, Writing – review & editing. PR: Conceptualization, Methodology, Supervision, Funding acquisition, Writing – review & editing. PM: Formal analysis, Writing - review & editing, Supervision.

## Footnotes

^1^Ge, Y., Correia, S., Fernandes, J., Hanson, K., Rato, A., and Rebuschat, P. (under review). Does phonetic training benefit word learning? Available at: https://osf.io/preprints/psyarxiv/5zspu.

^3^ www.prolific.com

^4^Among these additional languages, Cantonese and Shanghainese are tonal. Thus, we carried out an analysis to test whether the eight participants who spoke additional tonal languages performed differently from the others who did not know other tonal languages. However, adding *additional tonal experience* as a fixed effect in our model on CSWL accuracy did not significantly improve model fit (χ^2^(1) = 0, *p* = 1), nor did the 3-way interaction between block, additional tonal experience and trial type (χ^2^(7) = 11.177, *p* = 0.131). Thus, for the main analyses, we will not include additional tonal experience as a factor.

^5^HL experience was calculated from questions on frequency of HL use, for example, *how often do you speak it.* HL proficiency was based on questions such as *how well do you speak it.*

^6^Language entropy measures the level of language diversity in a particular context (e.g., family, external family, work, community, leisure) (Gullifer and Titone, 2020; Tomić et al., 2023). Higher language entropy in a given context means higher diversity in language use.

^7^ www.gorilla.sc
